# Pathways to understanding problem gambling among adolescents

**DOI:** 10.1186/s12889-025-23213-1

**Published:** 2025-06-10

**Authors:** Hyun Jung Lee, Gyungjoo Lee

**Affiliations:** 1 Department of Nursing, Jeonbuk Science College of Korea, Jeongeup, Republic of Korea; 2 College of Nursing, The Catholic University of Korea, 222 Banpo-daero, Seocho-gu, Seoul, 06591 Republic of Korea

**Keywords:** Addictive behaviors, Adolescent, Impulsivity, Gambling, Risk-taking

## Abstract

**Background:**

Gambling among youth is attracting the attention of health experts worldwide. A need has arisen for more research on the pathways to the development of adolescent problem gambling behavior and related factors.

**Methods:**

A cross-sectional study was conducted, and structure equation modeling was used to analyze data from the Korea Problem Gambling Agency to predict problem gambling among 1,474 adolescents aged 13–18 years.

**Results:**

Structural equation modeling showed that gambling accessibility and media exposure are associated with heightened sensation-seeking tendencies, which are related to stronger irrational gambling beliefs. The mediating associations of these factors play a significant role in predicting adolescent problem gambling. Additionally, ecological influences, including gambling accessibility and media exposure, show associations with the reinforcement of irrational gambling beliefs among adolescents. Although impulsivity was increased due to media exposure, it was not a significant mediator among gambling accessibility and media exposure, irrational gambling beliefs and problem gambling.

**Conclusion:**

Understanding adolescent problem gambling requires a comprehensive approach that considers both ecological and individual factors. To effectively reduce adolescent problem gambling, collaboration among researchers, policymakers, schools, parents, and adolescents is essential.

## Introduction

Problem gambling has been reported to have adverse effects on the well-being of adolescents [1, 2]. The prevalence of problem gambling among adolescents ranged from 0.7 to 12.3% across the world [1]. The UK Gambling Commission showed a decrease in adolescent problem gamblers from 1.7% in 2018 to 0.7% in 2023 [2, 3]. In South Korea, there was little change in the prevalence of adolescent’s problem gambling, which were 0.8% in 2018, 0.7% in 2020, and 0.8% in 2022 [4, 5]. However, the proportion of online gambling games involving betting money increased from 8.2% in 2018 to 11.7% in 2020 and 25.8% in 2022, with a particularly sharp rise reported in the experience rate of online gambling games among elementary school students [4, 5]. Even more serious is the trend of the age at which individuals begin gambling, with 6.5% starting at age 6 or below, 19.5% at ages 7–9, and 40.2% at ages 10–12[4]. Adolescents are likely to lose control and exhibit impulsivity more readily than adults [1]. The gambling problem manifests when they lose control over their gambling behavior, leading to excessive immersion in gambling to the extent that it interferes with daily activities and responsibilities, and causes harm [3]. Individuals who engage in gambling during early adolescence are at a higher risk for gambling addiction during adulthood [6, 7]. Many adolescents who become pathological gamblers become aware of the severity of the problem and begin to seek help only when they are in their 40s [8]. Problem gambling during adolescence most likely leads to depression, self-injury, and further addictive behaviors, which ultimately ruin adolescents’ lives [3, 9]. Therefore, more specific strategies should be developed to prevent problem gambling at an early developmental stage.

Blaszczynski and Nower posit gambling initiation is largely determined by the availability and accessibility of gambling opportunities, which serve as key ecological determinants [10]. Many studies have reported that increased accessibility to gambling opportunities correlates with higher rates of gambling addiction [11, 12]. In recent years, adolescent access to gambling has expanded significantly due to marketing strategies employed by online gaming companies targeting young consumers [13]. Additionally, exposure to gambling-related content through social networking services (SNS) and other online platforms has further increased gambling opportunities for adolescents [14, 15]. In South Korea, the proliferation of high-speed internet has created an environment highly conducive to gambling. The widespread availability and accessibility of legal gambling options, the rapid expansion of the gambling market, and the rise of illegal gambling platforms via smartphone apps and websites have all contributed to this trend [16, 17]. Moreover, adolescents exposed to gambling at an early age are more likely to have family members, peers, or acquaintances who engage in gambling activities. Such exposure suggests that many individuals become familiar with gambling through their social environment before actively participating themselves [3, 18]. Prior research underscores the critical role of gambling accessibility as a key ecological factor in understanding adolescent problem gambling.

Adolescence is characterized by an increased propensity for risky behaviors, largely driven by heightened sensation-seeking and impulsivity, which develop without a corresponding maturation in risk assessment abilities [18–20]. Key features of adolescence, such as impulsivity and sensation-seeking tendencies, have been identified as risk factors for gambling addiction among problem gamblers [21, 22]. Impulsivity is a well-established predictor of both the frequency and severity of problem gambling [23–25]. Additionally, adolescents with a strong sensation-seeking tendency are more likely to engage in recurrent gambling behavior [9]. Gambling itself further stimulates impulsivity and sensation-seeking, reinforcing continued gambling behavior [26]. However, research findings on sensation-seeking remain mixed. Some studies suggest that pathological gamblers exhibit lower sensation-seeking scores compared to control groups, raising questions about the role of sensation-seeking in gambling behaviors [27]. Given these inconsistencies, further research is needed to determine whether impulsivity and sensation-seeking should be considered key factors in addressing adolescent gambling problems.

Beyond sensation seeking and impulsivity, cognitive distortions play a crucial role in gambling behavior. [10]. Through repeated engagement in gambling, individuals develop irrational cognitive schemas related to gambling, such as distorted evaluations of gambling outcomes and unrealistic perceptions of winning probabilities [10]. These irrational gambling beliefs contribute to persistent gambling behavior despite repeated losses, as individuals falsely believe that a win is inevitable, even when they are aware of the low probability of such an occurrence [10]. Adolescents who hold these distorted beliefs are more likely to engage in gambling and develop an intention to continue gambling in the future, which directly increases their gambling behavior [28, 29]. Importantly, gambling accessibility is not limited to physical access to gambling platforms. Psychological accessibility shaped by permissive gambling environments within families and peer groups also plays a significant role in reinforcing gambling behavior [3, 18]. Such a socially permissive gambling environment can increase an adolescent’s likelihood of seeking excitement through gambling, [3, 18]. which in turn strengthens irrational gambling beliefs for winning probabilities and heightens vulnerability to problem gambling [29]. Irrational gambling beliefs among adolescents may serve as a key conditioning factor in the relationship between gambling accessibility, sensation-seeking, impulsivity, and problem gambling, highlighting the necessity of addressing these cognitive distortions alongside other risk factors in effective interventions.

**Fig. 1:**
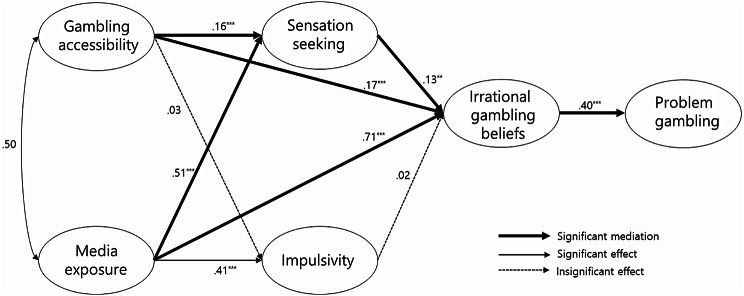
Final path diagram of the research model. The figure represents the beta values for each research path; ^*^p <.05, ^**^p <.01, ^***^p <.001

Blaszczynski and Nower’s Pathways Model of Problem and Pathological Gambling is a comprehensive framework that integrates biological, psychological, cognitive, and ecological factors to explain the onset and maintenance of gambling problems [10]. According to this model, impulsivity-related traits such as sensation seeking, attention-deficit/hyperactivity disorder (ADHD), and poor emotional regulation often underpinned by dopaminergic and serotonergic neurocircuitry form a key vulnerability pathway. Importantly, adolescence represents a neurodevelopmentally sensitive period characterized by heightened dopaminergic activity, increased sensation seeking, and immature self-regulation [19, 18, 20]. In this context, exposure to ecologically accessible gambling environments may further stimulate adolescents’ sensation-seeking tendencies and impulsivity, increasing the likelihood of adopting irrational gambling-related beliefs (e.g., illusion of control, gambler’s fallacy) and reinforcing habitual gambling behaviors. Such irrational gambling beliefs have been empirically shown to mediate the relationship between impulsivity, sensation seeking, and problem gambling, particularly among adolescents [21–23]. These interrelated ecological, psychological, and cognitive processes are hypothesized to mediate the risk for problem and pathological gambling in adolescence. Based on this framework and existing developmental literature, this study posits that ecological factors (e.g., gambling accessibility and media exposure) influence psychological traits (e.g., sensation seeking, aggression), which in turn contribute to cognitive distortions (e.g., biased evaluation, irrational beliefs), ultimately predicting the severity of problem gambling. (See Fig. 1.)

While previous research has largely examined ecological and psychological factors in adolescent problem gambling separately, more research is needed to explore their complex relationship. This study applied a comprehensive approach by investigating the intricate interplay between ecological, psychological, and cognitive factors and problem gambling within an integrated model. This study tested a structural equation model to predict adolescent problem gambling by incorporating gambling accessibility and media exposure as ecological determinants, along with sensation-seeking and impulsivity as psychological factors, and irrational gambling beliefs as cognitive factors.

## Methods

A cross-sectional descriptive design using secondary data was used to establish and test a structural equation model to predict problem gambling among adolescents. The study was approved by the Institutional Review Board of our institution.

### Data

Raw data used in this study were obtained from ‘*A Model to Prevent Problem Gambling among Adolescents based on the Protective and Risk Factors’*, conducted by the Korea Center on Gambling Problems (KPGA) from September to December 2017 [30]. Data were collected from 1,536 adolescents aged 13 to 18 through a nationwide online and offline survey conducted by a research firm. From the initial sample of 1,536 adolescents aged 13–18 years, data from 1,474 adolescents attending school were examined after excluding 60 datasets from out-of-school adolescents and datasets of two boys with missing values for study variables. The sample size estimated for this study was n = 1,474 indicating that the recommended number for an analysis with a structural equation model was achieved. Table 1 presents the descriptive data of study variables.

**Table 1:** Descriptive statistics of study variables by problem gambling severity (*N* = 1,474)

Variables	Categories	*n* (%) or mean ± SD			χ^2^or F(*p*) Scheffe
		No problem 1392(94.4)	Low-to-moderate severity 53(3.6)	High severity 29(2.0)	
Gender	Male	608 (92.5)	30 (4.6)	19 (2.9)	8.70(0.013)
	Female	784 (96.0)	23 (2.8)	10 (1.2)	
Age (years)		16.55 ± 1.24	16.64 ± 1.16	16.93 ± 1.28	1.40 (0.247)
Type of school	Middle school	311 (96.0)	8 (2.5)	5 (1.5)	1.95(0.377)
	High school	1081 (94.3)	45 (3.9)	24 (2.1)	
Monthly allowance	< 50,000	539 (96.9)	13 (2.3)	4 (0.7)	11.58(0.003)
(KRW)	≥ 50,000	853 (92.9)	40 (4.4)	25 (2.7)	
Living arrangement	Living with parents	1333 (94.8)	49 (3.5)	24 (1.7)	12.32(0.015)
	Living with relatives	10 (83.3)	1 (8.3)	1 (8.3)	
	Other	49 (87.5)	3 (5.4)	4 (7.1)	
Perceived economic Status	High	378 (94.5)	12 (3.0)	10 (2.5)	5.47(0.242)
	Middle	691 (95.3)	22 (3.0)	12 (1.7)	
	Low	323 (92.6)	19 (5.4)	7 (2.0)	
Gambling accessibility		1.54 ± 0.74^a^	2.42 ± 0.89^b^	3.26 ± 0.84^c^	107.20 (< 0.001) a < b < c
Media exposure		1.95 ± 0.73^a^	2.73 ± 0.82^b^	3.49 ± 0.81^c^	88.55(< 0.001) a < b < c
Sensation-seeking		2.77 ± 0.94^a^	3.14 ± 1.01^b^	3.76 ± 0.73^c^	16.23(< 0.001) a < b < c
Impulsivity		2.22 ± 0.79^a^	2.55 ± 0.75^b^	2.95 ± 1.05^c^	18.96(< 0.001) a < b,c
Irrational gambling belief		1.94 ± 0.68^a^	2.64 ± 0.59^b^	3.18 ± 0.84^c^	71.02(< 0.001) a < b < c

Note: SD, standard deviation; SNS, social networking services

### Study variables

The Adolescent Problem Behavior Protective-Risk Factors Scale, developed and validated by the KPGA, originally consisted of 270 items categorized into personal, family, peer, school, and community domains related to protective and risk factors for adolescent gambling. In this study, only items related to media exposure, sensation seeking, impulsivity, irrational gambling beliefs, and gambling accessibility were used. To confirm the suitability of the factor structure for each variable in the current sample, separate exploratory factor analyses (EFAs) were conducted using principal component analysis with varimax rotation. The number of subfactors and cumulative explained variance for each construct were as follows: one factor (69.06%) for gambling accessibility, two factors (73.84%) for media exposure, two factors (61.49%) for sensation seeking, one factor (59.95%) for impulsivity, and three factors (68.13%) for irrational beliefs. Based on the EFA results, one item from the media exposure scale and two items from the irrational beliefs scale with factor loadings below 0.60 were removed. A confirmatory factor analysis (CFA) was then conducted on the remaining items to assess the overall measurement model. The model showed a good fit to the data (TLI = 0.91, CFI = 0.96, RMSEA = 0.05).

*Gambling accessibility* was evaluated using 10 items regarding the accessibility of the place, the methods, and fundraising for gambling [30]. Sample questions included “I can gamble from home if I want to.” and “I can gamble when hanging out with friends.” The responses were scored on a 5-point Likert scale ranging from 1 (“not at all”) to 5 (“very much so”), with a higher score indicating higher levels of gambling accessibility. Cronbach’s α was 0.95 in this study.

*Media exposure* was evaluated using seven items regarding the experience of online and offline gambling advertising [30]. The sample questions included: “I have accessed gambling websites through SNS advertisements” and “I have encountered advertisements on SNS claiming easy access to loans.” The responses were scored on a 5-point Likert scale ranging from 1 (“not at all”) to 5 (“very much so”), with a higher score indicating higher levels of media exposure. Cronbach’s α was 0.78 in this study.

*Sensation-seeking* was evaluated using eight items regarding the desire to pursue an experience of thrill and adventures [30]. The sample items included “I want to experience something thrilling, even if it scares me.“and “I want to try bungee jumping.” The responses were scored on a 5-point Likert scale ranging from 1 (“not at all”) to 5 (“very much so”), with a higher score indicating higher levels of sensation-seeking. Cronbach’s α was 0.83 in this study.

*Impulsivity* was evaluated using four items regarding negative urgency [30]. The sample item included “When angry, I yell or throw objects.” The responses were scored on a 5-point Likert scale ranging from 1 (“not at all”) to 5 (“very much so”), with a higher score indicating higher levels of impulsivity. Cronbach’s α was 0.78 in this study.

*Irrational gambling beliefs* was evaluated using 13 items regarding the distorted cognitive expectations of the gambling process and results [30]. The sample items included “Gambling is a better way to make money than working.” and “I always predict gambling outcomes better than other people.” The responses were scored on a 5-point Likert scale ranging from 1 (“not at all”) to 5 (“very much”), with a higher score indicating higher levels of irrational gambling beliefs. Cronbach’s α was 0.91 in this study.

*Problem gambling* was evaluated using nine items on the General Problem Severity Subscale (GPSS) of the Canadian Adolescent Gambling Inventory [31]. The GPSS is a scale that selects 9 items from the CAGI’s 24 items developed by Tremblay et al. [31]. to estimate the level of adolescent gambling problems in the past 3months. The KPGA data we used included questions about gambling experiences in the past year. A sample item is “How often have you skipped practice or dropped out of activities (such as team sports or band) due to your gambling?” The responses were scored on a 4-point Likert scale ranging from 0 (“never”) to 3 (“almost always”). Scores ranged from 0 to 27; scores 0–1 indicated “No problem gambling” (Green light), 2–5 indicated “Low to moderate severity” (Yellow light), and 6 indicated “High severity” (Red light). Green light indicates a state where there has been no gambling experience, or any harm or damage caused by gambling. The yellow light indicates that there has been gambling experience and there are suspicions of progressing to a problem level with failures in control ranging from low to moderate levels. In red light, there have been repeated gambling experiences and failures in control have reached a severe level. It is interpreted as a high risk of gambling addiction. Cronbach’s α was 0.89 in this study.

### Data analysis

The data collected in this study were analyzed using IBM SPSS Statistics for Windows version 18.0 (IBM Corp., Armonk, NY, USA) and AMOS 21.0 (IBM Chop.). Descriptive statistics were obtained regarding the participants’ general characteristics and measured variables. Normality was tested using the skewness and kurtosis values. Pearson’s correlation coefficients were used to analyze the correlations across the measured variables, as well as Cronbach’s α. We further employed the maximum likelihood method to validate the structural equation model. A confirmatory factor analysis was performed to evaluate the validity of the instrument for the latent variables. To assess the fit of the hypothetical model, the following indices were used: χ2, df, Root Mean Square Error of Approximation (RMSEA), Tucker-Lewis Index (TLI), and Comparative Fit Index (CFI). To test the significance of the pathways in the revised and hypothetical models, standardized estimates, critical ratios, and p values were used. To estimate the explanatory power of endogenous variables, squared multiple correlation was used. To test the significance of the direct, indirect, total, and mediating effects of the model, bootstrapping was used because problem gambling failed to satisfy multi-variate normality [32].

## Results

### Descriptive statistics and correlations across study variables

Table 1 presents the mean and standard deviation for each study variable. The skewness and kurtosis in this study were -1.22-51.72 and − 0.23‒6.63, respectively; normality was satisfied by all variables except problem gambling severity. Bootstrapping was used because problem gambling failed to satisfy multi-variate normality. The correlations across the latent variables in this study fell at or below 0.70, suggesting that the correlations across independent variables were not high, indicating the absence of a multicollinearity problem. All variables were found to be significantly correlated (Table 2).

**Table 2:** Correlation coefficient between variables

Variables	Gambling accessibility *r* (*p*)	Media exposure *r* (*p*)	Sensation-seeking *r* (*p*)	Impulsivity *r* (*p*)	Irrational gambling beliefs *r* (*p*)
Gambling accessibility	–				
Media exposure	0.37 (< 0.01)	–			
Sensation-seeking	0.32 (< 0.01)	0.42(< 0.01)	–		
Impulsivity	0.21 (< 0.01)	0.26 (< 0.01)	0.33 (< 0.01)	–	
Irrational gambling beliefs	0.43 (< 0.01)	0.53 (< 0.01)	0.50 (< 0.01)	0.34 (< 0.01)	–
Problem gambling	0.35 (< 0.01)	0.26 (< 0.01)	0.23 (< 0.01)	0.14 (< 0.01)	0.26(< 0.01)

All correlations were *p* <.01

### Parameter Estimation and effect analysis of the research model

All factors showed convergent validity with critical ratio (C.R.) above 1.96, average variance extracted above 0.50, and construct reliability (CR) above 0.70. The goodness of fit of the hypothetical model was TLI 0.83, CFI 0.87, and RMSEA 0.08. The final research model was established using a Modification Index of 10.0 or higher as a modification alternative. The fit indices for the final research model were χ^2^ = 373.69 (df = 54, *p* <.001), RMSEA = 0.06, TLI = 0.93, and CFI = 0.95. Table 3 presents the significance, and standardized parameter estimates of pathways for the model. To test the significance and determine the structural relationship for the model in this study, bootstrapping was used to analyze the effects. Gambling accessibility and media exposure showed direct effect on sensation-seeking (β = 0.16, *p* =.006; β = 0.51, *p* =.003) and irrational gambling belief (β = 0.17, *p* =.018; β = 0.71, *p* =.004). Sensation-seeking showed direct effect on irrational gambling beliefs (β = 0.13, *p* =.002) and irrational gambling beliefs showed direct effect on problem gambling (β = 0.40, *p* =.002). Media exposure showed direct effect on impulsivity (β = 0.41, *p* =.002), but direct effect of gambling accessibility on impulsivity and direct effect of impulsivity on irrational gambling beliefs were not significant (β = 0.03, *p* =.471; β = 0.02, *p* =.480; Table 3; Fig. 1).

**Table 3:** Direct, indirect, and total effects for the research model

Endogenous variables	Exogenous variables	β (SE)	C.R. (*p*)	SMC	Direct	Indirect	Total
					β (*p*)	β (*p*)	β (*p*)
Sensation-seeking	Gambling accessibility	0.16 (0.03)	3.80 (< 0.001)	0.38	0.16 (0.006)		0.16 (0.006)
	Media exposure	0.51 (0.05)	8.45 (< 0.001)		0.51 (0.003)		0.51 (0.003)
Impulsivity	Gambling accessibility	0.03 (0.03)	0.80 (0.423)	0.19	0.03 (0.471)		0.03 (0.471)
	Media exposure	0.41 (0.05)	8.17 (< 0.001)		0.41 (0.002)		0.41(0.002)
Irrational gambling beliefs	Gambling accessibility	0.17 (0.04)	3.38 (< 0.001)	0.81	0.17 (0.018)	0.02 (0.012)	0.19 (0.017)
	Media exposure	0.71 (0.08)	9.31 (< 0.001)		0.71 (0.004)	0.07 (0.019)	0.78 (0.002)
	Sensation-seeking	0.13 (0.06)	2.84 (0.004)		0.13 (0.002)		0.13 (0.020)
	Impulsivity	0.02 (0.03)	0.70 (0.485)		0.02 (0.480)		0.02 (0.480)
Problem gambling	Gambling accessibility					0.08 (0.014)	0.08 (0.014)
	Media exposure					0.31 (0.002)	0.31 (0.002)
	Sensation-seeking					0.05 (0.019)	0.05 (0.019)
	Impulsivity					0.01 (0.459)	0.01 (0.459)
	Irrational gambling beliefs	0.40 (0.01)	14.35 (< 0.001)	0.16	0.40 (0.002)		0.40 (0.002)

Note. β, Standardized coefficient; SE, Standard error; C.R., Critical ratio; SMC, Squared multiple correlation

### Mediating effects in the model

The mediating effects were analyzed using phantom variables for the model (Table 4; Fig. 1). Gambling accessibility (B < 0.01, *p* =.012) and media exposure (B = 0.01, *p* =.015) increased problem gambling severity by the double mediation of sensation-seeking and irrational gambling beliefs. Gambling accessibility (B = 0.03, *p* =.011) and media exposure (B = 0.14, *p* =.002) increased problem gambling mediated by irrational gambling beliefs. The double mediation effect of impulsivity and irrational gambling beliefs on the relationship between gambling accessibility and problem gambling severity was not significant (B < 0.01, *p* =.425). The double mediation effect of impulsivity and irrational gambling beliefs on the relationship between media exposure and problem gambling severity was not significant (B = 0.02, *p* =.435).

**Table 4:** Mediating effects of the research model

Independent variables	Mediating variables	Dependent variables	B	*p*
Gambling accessibility	→Sensation-seeking	→ Irrational gambling beliefs	0.02	0.013
Gambling accessibility	→ Sensation-seeking → Irrational gambling beliefs	→ Problem gambling	0.00	0.012
Gambling accessibility	→ Impulsivity	→ Irrational gambling beliefs	0.00	0.410
Gambling accessibility	→ Impulsivity→ Irrational gambling beliefs	→ Problem gambling	0.00	0.425
Gambling accessibility	→ Irrational gambling beliefs	→ Problem gambling	0.03	0.011
Media exposure	→ Sensation-seeking	→ Irrational gambling beliefs	0.07	0.020
Media exposure	→ Sensation-seeking → Irrational gambling beliefs	→ Problem gambling	0.01	0.015
Media exposure	→ Impulsivity	→ Irrational gambling beliefs	0.01	0.417
Media exposure	→ Impulsivity→ Irrational gambling beliefs	→ Problem gambling	0.02	0.435
Media exposure	→ Irrational gambling beliefs	→ Problem gambling	0.14	0.002
Sensation-seeking	→ Irrational gambling beliefs	→ Problem gambling	0.03	0.020
Impulsivity	→ Irrational gambling beliefs	→ Problem gambling	0.00	0.468

Note. B, Unstandardized coefficient

## Discussion

This study aimed to test a structural equation model based on Blaszczynski and Nower’s pathways model [10]. of problem and pathological gambling to predict problem gambling among adolescents in South Korea. The main findings demonstrated that gambling accessibility and media exposure predicted problem gambling through the dual mediation of sensation seeking and irrational gambling beliefs. In adolescents, gambling accessibility and media exposure are associated with increased sensation-seeking, which, in turn, contributes to heightened irrational gambling beliefs. The mediational relationships among these variables significantly predict adolescent problem gambling. Furthermore, ecological factors, such as gambling accessibility and media exposure, are directly linked to an increase in irrational gambling beliefs. These findings support the assumptions of Blaszczynski and Nower’s Pathways Model of Problem and Pathological Gambling, which posits that gambling develops into a gambling problem through the conditioning of cognitive schemas driven by the relationship between the psychological factor associated with sensation-seeking, such as a preference for thrill and adventure, and exposure to accessible gambling environments.

The present study found that gambling accessibility and media exposure are positively associated with heightened irrational gambling beliefs among adolescents. These findings align with previous studies that reported the impact of gambling accessibility on irrational gambling beliefs among adolescents [12, 26]. Additionally, the results are consistent with Kim et al.’s findings, which indicate that exposure to sports betting advertisements increases irrational gambling beliefs among university male students [33]. Po Oei et al. [34] suggested that the persistence of problematic gambling behavior stems from individuals’ erroneous beliefs and misconceptions about their ability to control or predict gambling outcomes [34]. Adolescents are cognitively and developmentally immature, making them more sensitive to marketing campaigns and thus more vulnerable to gambling-related issues [3]. Adolescents exposed to marketing advertisements by gambling companies designed to encourage gambling are more likely to initiate gambling. Such advertisements may lead adolescents to believe that they can easily win money and achieve social success through gambling [35]. Adolescents reported encountering gambling for the first time through exposure to illegal online gambling advertisements [36]. Online gambling advertisements, unrestricted by time and space, expose adolescents to various forms of gambling, making access easier and more problematic [37]. Early initiation of gambling during adolescence may be attributed to risky ecological factors such as high online gambling accessibility and unregulated media exposure. These ecological risk factors directly increase irrational gambling beliefs and may worsen adolescents’ gambling-related problems.

The present study suggests that a gambling-accessible ecological environment is not only directly associated with heightened gambling-related beliefs but also indirectly contributes to the formation of irrational gambling beliefs among adolescents through its relationship with sensation seeking. Positive sensation-seeking experiences, such as the thrill and adventure associated with gambling, encourage gambling engagement [21]. Such stimulating experiences increase the likelihood of continued sensation seeking and contribute to the development of cognitive biases related to gambling success [38]. Research on adults has shown that internet gambling advertisements and promotions lead to increased gambling consumption among existing gamblers or facilitate a shift from offline to online gambling, without necessarily creating new gamblers [39]. However, with the growing accessibility of the internet, adolescents are now more likely to encounter gambling online for the first time [36]. Online gambling advertisements, which often depict gambling as an easy way to win money, can stimulate sensation-seeking tendencies in neurologically immature adolescents, drawing them into deeper gambling involvement. Additionally, the portrayal of sports betting as a typical and entertaining activity in online marketing can lead adolescents to perceive it as a harmless game rather than a form of gambling. [40]. Furthermore, adolescents’ first winning experiences in gambling can heighten sensation-seeking and reinforce irrational beliefs about gambling success. In this study, adolescents in the problem gambling group showed higher levels of sensation-seeking related to thrill and adventure compared to adolescents in the normal group. Notably, Blaszczynski’s earlier research on adult male pathological gamblers reported lower scores on thrill and adventure scales, [27]. whereas previous studies on adolescents with problem gambling indicated higher sensation-seeking within this group [41]. This discrepancy suggests that sensation-seeking tendencies may play a more prominent role in adolescent gambling behavior compared to adults. Adolescence is a developmental stage characterized by increased sensation-seeking and underdeveloped self-regulation, both of which contribute to a greater tendency toward risk-taking behaviors [18, 20]. This inclination can be shaped by both biological and social influences that encourage engagement in risky activities [18]. Given these theoretical insights, this study suggests that a gambling-accessible ecological environment can stimulate adolescents’ sensation-seeking tendencies, increase their acceptance of gambling, and foster irrational beliefs about gambling success ultimately exacerbating gambling-related problems.

In this study, adolescents classified as problem gamblers exhibited significantly higher levels of impulsivity and cognitive distortion compared to non-problem gamblers. Additionally, increased media exposure was associated with heightened impulsivity; however, impulsivity was not identified as a contributing factor to the development of enhanced irrational beliefs about gambling. Impulsivity is increasingly recognized as a complex and multidimensional construct, considered a heterogeneous set of traits encompassing various tendencies or behavioral inclinations [42]. In Michalczuk et al.‘s study, [43]. which examined the relationship between impulsivity and cognitive distortions, it was found that among the five subfactors of impulsivity negative urgency, positive urgency, lack of planning, lack of perseverance, and sensation-seeking only positive urgency was associated with the total score of cognitive distortions. Other researchhas also indicated that positive urgency is associated with cognitive distortions [34], as the expectation that gambling will be exciting and alleviate negative effects when in a good mood may further stimulate cognitive distortions. In our study, impulsivity was assessed briefly with four items, none of which reflected positive urgency. However, many studies have identified impulsivity as a predictor variable for problem gambling [25, 43, 41]. Further research incorporating the multidimensional aspects of impulsivity is needed, particularly focusing on adolescent populations.

Unlike prior studies that have predominantly considered ecological and personal factors in adolescent problem gambling independently, this research adopts a holistic perspective. By examining the dynamic interplay among gambling accessibility, media exposure, sensation-seeking, impulsivity, and irrational gambling beliefs within an integrated framework, this study provides a more comprehensive understanding of problem gambling among adolescents. This study provides evidence that exposure to a gambling-accessible environment can stimulate adolescents’ sensation-seeking tendencies, increasing the likelihood of perceiving gambling as an enjoyable and exciting activity or developing irrational gambling beliefs, such as the expectation of consistent wins based on initial winning experiences. These beliefs, in turn, may contribute to the development of problem gambling among adolescents. Addressing such issues cannot be resolved solely through individual-level interventions. The interaction between advertising and the gambling industry is complex, and more in-depth research is required to clarify the causal relationship with gambling behavior. Considering the continuous expansion of the online gambling industry and the advancement of advertising strategies, researchers and policymakers must work collaboratively to address these issues [35]. At the governmental level, a legal, policy, regulatory, and monitoring framework for the gambling industry should be established. Based on this framework, a collaborative system can be developed at the local level to intervene in adolescent gambling problems through cooperation between schools, parents, and other stakeholders. Furthermore, It is essential to develop and implement early preventive education and programs aimed at regulating ecological temptations related to gambling among adolescents.

This study has several limitations. First, as it involves secondary data analysis of KPGA data, which were derived from a cross-sectional study, the causal relationships among the study variables should be interpreted with caution. A longitudinal study is needed to examine time-dependent changes while considering the findings of this study. Second, the measures used in this study were initially developed and validated in the KPGA study, with further validation conducted in accordance with the model of this study. However, since these measures have not been widely used in various studies, it cannot be asserted that they effectively describe the conceptual construct of the variables used. Specifically, further research using more validated tools is necessary, particularly those that account for the structure and subdomains of sensation seeking and impulsivity. Third, the data used in this study were self-reported by adolescents, which introduces the possibility of perception bias, recall errors, or unreliable responses. Additionally, while differences in various variables were presented based on the level of problem gambling, there may be potential confounding factors related to regular gamblers and regular problem gamblers. Moreover, owing to the low proportion of at-risk and problem gamblers and the large dispersion, the model’s explanatory power was limited. Lastly, the data for this study were collected prior to the occurrence of COVID-19 and, therefore, do not reflect the changes resulting from the pandemic’s impact.

## Conclusion

This study highlights that greater gambling accessibility and media exposure are associated with heightened sensation-seeking tendencies, which are related to stronger irrational gambling beliefs. The mediating associations of these factors play a significant role in predicting adolescent problem gambling. Additionally, ecological influences, including gambling accessibility and media exposure, show associations with the reinforcement of irrational gambling beliefs among adolescents. Effectively tackling these issues necessitates close cooperation between researchers and policymakers, given the intricate relationship between advertising and the gambling industry. Establishing a government-driven legal and regulatory framework is essential, alongside localized initiatives that engage schools, parents, and community stakeholders to identify and assist at-risk adolescents. To mitigate adolescent gambling problems, it is crucial to implement preventive strategies that holistically account for both ecological and individual influences. 

## Data Availability

The secondary data used in this study are available on request from KPGA according to the regulations of the KPGA raw data use. The qualitative datasets generated and/or analysed during the current study are not publicly available due to privacy restriction, but are available from the corresponding author on reasonable request.
